# Antibody response induced by structural proteins from *Triatoma virus* as potential adjuvants in experimental immunisation models

**DOI:** 10.3389/fmicb.2026.1753585

**Published:** 2026-03-03

**Authors:** Aline Maria Vasconcelos Queiroz, Annamairlla do Nascimento Oliveira, Alzira Regina Silva de Deus, Ingrid Emiliane Fonseca de Oliveira, Isaías Amâncio dos Santos, Fred Luciano Neves Santos, Paola Alejandra Fiorani Celedon, Diego M. A. Guérin, Ana Rosa Viguera, Marcelo Sousa-Silva

**Affiliations:** 1 Postgraduate Programme in Pharmaceutical Sciences, Federal University of Rio Grande do Norte, Natal, Brazil; 2 Department of Clinical and Toxicological Analysis, Immunoparasitology Laboratory, Federal University of Rio Grande do Norte, Natal, Brazil; 3 Advanced Public Health Laboratory, Gonçalo Moniz Institute, Fiocruz, Salvador, Brazil; 4 Carlos Chagas Institute, Fiocruz, Curitiba, Brazil; 5 Instituto Biofisika (UPV/EHU, CSIC), Leioa, Spain; 6 Global Health and Tropical Medicine, Institute of Hygiene and Tropical Medicine, Universidade Nova de Lisboa, Lisbon, Portugal

**Keywords:** adjuvant, humoral immune response, immunisation models, recombinant proteins, *Triatoma virus*, vaccine, virus-like particles (VLPs)

## Abstract

**Introduction:**

Virus-Like Particles (VLPs) are viral protein structures widely used as adjuvants in vaccine formulations due to their ability to stimulate the innate immune response, thereby contributing to the activation of adaptive immunity through the production of different IgG subclasses. The present study evaluated the adjuvant potential of recombinant structural proteins of the *Triatoma virus* VLPs (TrV-VLPs: VP1, VP2, and VP3) in experimental immunisation protocols for American Cutaneous Leishmaniasis and Chagas disease.

**Methods:**

BALB/c mice were immunised with native antigens of Leishmania amazonensis or chimeric recombinant antigens of *Trypanosoma cruzi* in association with different adjuvants, including aluminium hydroxide, incomplete Freund’s adjuvant, and VPs structural proteins. The induction of specific antibodies (anti-*L. amazonensis* or anti-recombinant proteins of *T. cruzi*) was measured by ELISA to determine the IgG subclass profile.

**Results and Discussion:**

Immunisation with *L. amazonensi*s antigens revealed that VPs preferentially induced IgG2b and IgG3 antibodies, whereas in experiments with *T. cruzi* antigens, IgG2b was predominant, accompanied by similar levels of IgG2a and IgG3, compared to lower IgG1 responses. These findings suggest that recombinant structural proteins of TrV-VLPs represent a promising adjuvant strategy capable of modulating humoral immune responses, offering potential applications in vaccine development against protozoan parasites such as Leishmania spp. and *T. cruzi*.

## 1 Introduction

Vaccine adjuvants are substances which improve the immunogenicity of a given antigen during an immunisation process. Therefore, they are important components for improving the efficacy of vaccines, mainly due to the increase in the speed, duration, and robustness of the immune response induced during the immunisation process. Consequently, vaccine adjuvants are important for improving the immune response in immunologically compromised individuals and reducing the antigen to be used in vaccine preparation (Facciolà et al., 2022).

Historically, the first licencing of adjuvant occurred in the mid-1920s. Several vaccines were subsequently developed using alum adjuvants in different vaccine preparations, such as vaccines for hepatitis B, diphtheria, tetanus, pertussis, or human papillomavirus. It was the only adjuvant included in licenced products for 70 years (Zhao et al., 2023a). It was not until the 1990s that new adjuvants emerged and had their efficacy and safety approved for inclusion in licenced products, such as the oil-in-water emulsion adjuvant MF59, a trivalent inactivated seasonal influenza vaccine specifically designed for adults over 65 years old (Pulendran et al., 2021). Even though many adjuvants have shown great efficacy in preclinical models over time, most have not yet been licenced for use in humans, usually due to safety or tolerability issues (Stewart et al., 2022; Wang et al., 2019), making it essential to encourage research aimed at the discovery and development of safe and effective adjuvants.

Recombinant proteins have played a crucial role in the production of modern vaccines. Through recombinant DNA technology, proteins of specific genes from the target pathogen are produced in host cells such as bacteria, yeasts, insect cells, and mammalian cells (Cid and Bol, 2021). This vaccine concept is effective because the produced protein mimics the pathogen’s antigen, stimulating the immune system to generate a specific response (Pollet et al., 2021).

Another significant advancement in the field of vaccines is the use of VLPs, which in recent years have been extensively used as adjuvants. VLPs consist of organised structural viral proteins (VPs) which do not contain viral genetic material (Zhang et al., 2023), but retain the general structure, allowing them to display some PAMPs present in the native virus. They can be produced in different expression systems, such as bacteria, yeasts, baculoviruses, insect cells, mammalian cells, plant cells, and cell-free systems. Some examples of the use of the VLPs platform in vaccine development include the hepatitis B vaccine, which has been in use since the 1990s (Gupta et al., 2023).

Infections caused by species of the *Leishmania* genus have global distribution, occurring in 98 countries and in all regions of the World Health Organization (WHO), with a population of 600 million to 1 billion people at risk of infection (Coutinho De Oliveira et al., 2020). In the specific case of ACL, also known as New World Cutaneous Leishmaniasis (CL), the number of people at risk ranges from 1 billion to 399 million. In the context of vaccines, recent studies have focused on developing a chimeric influenza VLPs expressing the surface antigen of *L. amazonensis* promastigotes (LaPSA-VLPs) administered in two doses subcutaneously (Eom et al., 2024); on the exploration of a two-dose oral immunisation strategy using heat-inactivated *Mycobacterium bovis* (HIMB) (Ferreras-Colino et al., 2023); as well as nanoparticle-based formulations associated with *L. amazonensis* antigens for protection against visceral leishmaniasis (VL) (González et al., 2023).

Chagas disease is caused by the protozoan *T. cruzi*, and is amongst the 20 neglected tropical diseases recognised by the WHO. It is estimated that 70 million people worldwide are at risk of this infection, whilst those affected by the disease vary between 6 and 7 million (WHO, 2024). In the vaccine scenario, the CRUZIVAX project is under development in three South American countries (Argentina, Bolivia, and Paraguay), which is conducting preclinical studies using the Transpain antigen in combination with the cdiAMP adjuvant (Sanchez Alberti et al., 2017; Sanchez Alberti et al., 2020) to prevent and treat infection caused by *T. cruzi*. In addition, the project emphasises the relevance of health economics for advancing a vaccine and the consequent improvement in the quality of life of the affected populations (Siclla Godoy and Carnovale, 2024).

More recently, our group developed recombinant VLPs from *Triatoma virus* (TrV-VLPs) composed of three VPs (VP1, VP2, and VP3), and used them in a murine immunisation protocol (Queiroz et al., 2021a). TrV-VLPs were tested alone, in association with alum, or combined with chimeric recombinant antigens of *T. cruzi* (IBMP-8.1, IBMP-8.2, IBMP-8.3, and IBMP-8.4). The results demonstrated that the antibody profile for TrV-VLPs was similar (Total IgG, IgG1, IgG2a, and IgG2b) or higher (IgG3) than that observed with alum, even 70 days after administration of the second dose. When combined with *T. cruzi* antigenic candidates, it was observed that depending on the antigen used, TrV-VLPs did not induce IgG1 production (IBMP-8.1, IBMP-8.3 and IBMP-8.4), whilst IgG3 induction was superior (IBMP-8.1 and IBMP-8.2) or equivalent (IBMP-8.3) to the response obtained with alum. Given these results, we suggest that vaccines based on VLPs represent a promising technology for combating diseases caused by trypanosomatids, such as leishmaniasis, Chagas disease, and African trypanosomiasis (Queiroz et al., 2021b). Thus, the aim of this study was to evaluate the immunogenic capacity of vaccine formulations containing native antigens of *L. amazonensis* or chimeric recombinant antigens of *T. cruzi* (IBMPs), associated or not, with known adjuvants (FIA; alum) and to investigate the immunoadjuvant capacity of three recombinant structural proteins of TrV-VLPs (VP1, VP2 and VP3) regarding humoral immunity induced in a BALB/c mouse model.

## 2 Results

### 2.1 Electrophoretic profile of *Triatoma virus* structural proteins (VP1, VP2 and VP3)

**Figure 1:**
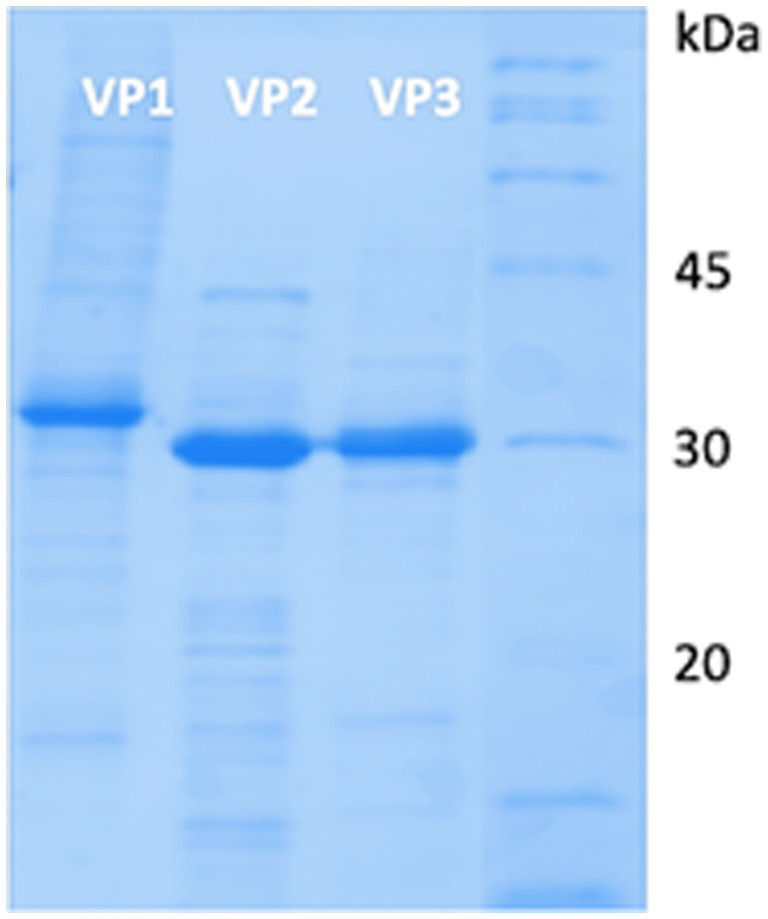
Electrophoresis gel (SDS-PAGE) of VP1, VP2, and VP3 recombinant structural proteins of TrV-VLPs.

Figure 1 illustrates the electrophoretic profile of the VP1, VP2, and VP3 recombinant structural proteins of TrV-VLPs. The profile was obtained by electrophoresis in a 12.5% polyacrylamide gel (SDS-PAGE), stained with Coomassie blue. The results reveal a protein profile characterised by several proteins with different molecular masses, with a major band around 30 kDa which suggests a higher concentration of this specific protein, corresponding to VP1, VP2 and VP3, respectively.

### 2.2 Anti-*leishmania* antibody profile in animals immunised with total protein extract of *L. amazonensis* and with different vaccine adjuvants

**Figure 2:**
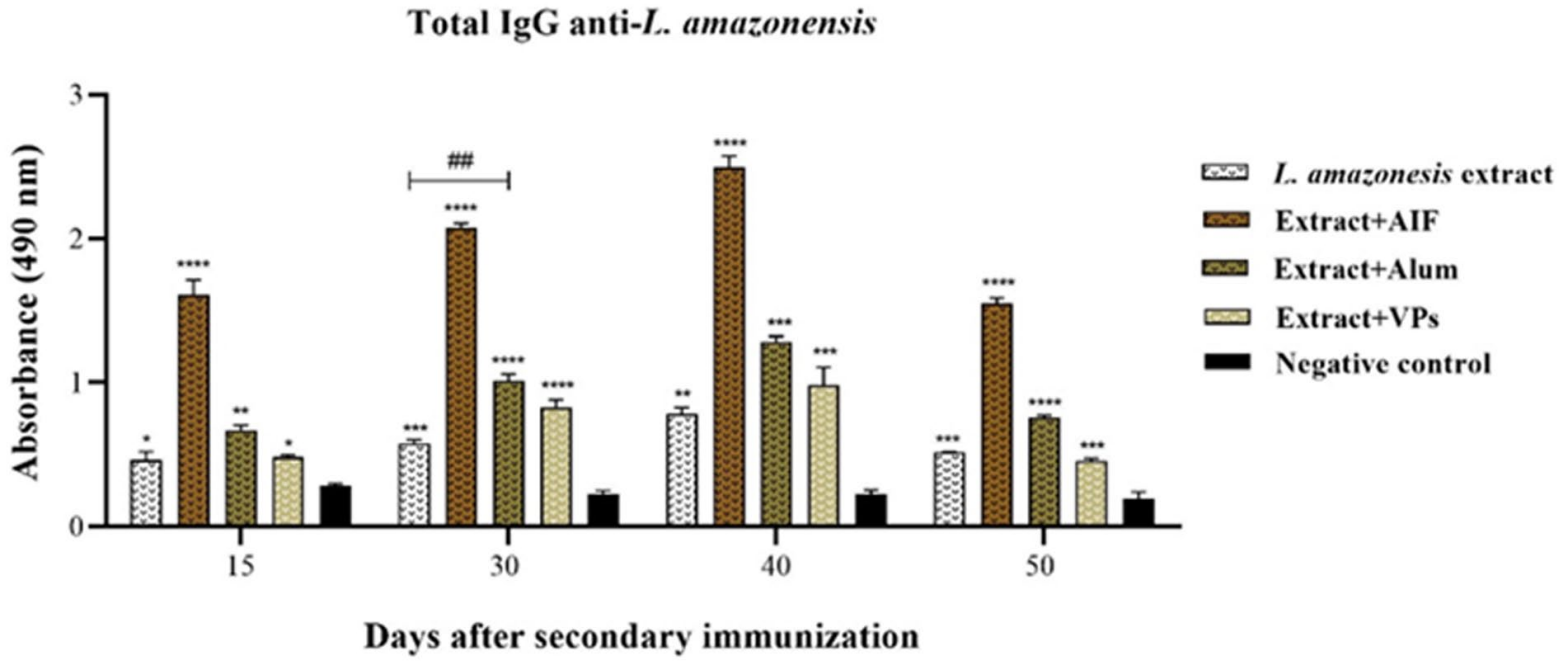
Determination of total IgG anti-*L. amazonensis* antibodies in animals immunised subcutaneously. The white background bars show the antibody profile of the immunised group only with the crude protein crude protein extract of *L. amazonensis*, whilst the other bars represent the immunised group with the crude protein extract in the presence of FIA, alum, and structural virus proteins (VPs). The antibody profile of non-immunised animals (negative control) is represented by the black bar. The antibody levels are measured by ELISA using a crude protein extract of *L. amazonensis*. Each bar represents the mean absorbance obtained in each group of immunised animals and their respective standard deviations 15 and 30 days post-immunisation. Serum samples were analysed at a dilution of 1:25 (v/v). Absorbance at 490 nm is shown on the *Y*-axis. Differences between the mean values were considered significant when *p* < 0.05. The symbols “*” and “#” indicate the differences observed in the immunised groups compared to the negative control and the difference between the groups immunised only with crude protein extract vs. crude protein extract mixed with VPs.

Serum samples from immunised mice at 15 and 30 days post-immunisation were used and compared with non-immunised mice (negative control) in order to determine the level of anti-*Leishmania* IgG antibodies, after the primary immunisation in mice by injection of total antigens of *L. amazonensis* formulated with the different vaccine adjuvants (alum, FIA, and VPs). The antibody profile was assessed against the native antigens of *L. amazonensis* by ELISA. The results presented in Figure 2 show that an increase in total IgG anti-*L. amazonensis* antibody levels was observed in all immunised groups 15 days after the first dose when compared to the negative control. All immunised groups (FIA, alum, and VPs) showed similar antibody profiles, with no significant difference between them. However, a statistically significant difference was found when comparing these three groups with the group immunised only with *L. amazonensis* crude protein extract. Additionally, the total IgG anti-*L. amazonensis* antibody levels in the immunised groups remained elevated 30 days after the first immunisation compared to the negative control.

**Figure 3:**
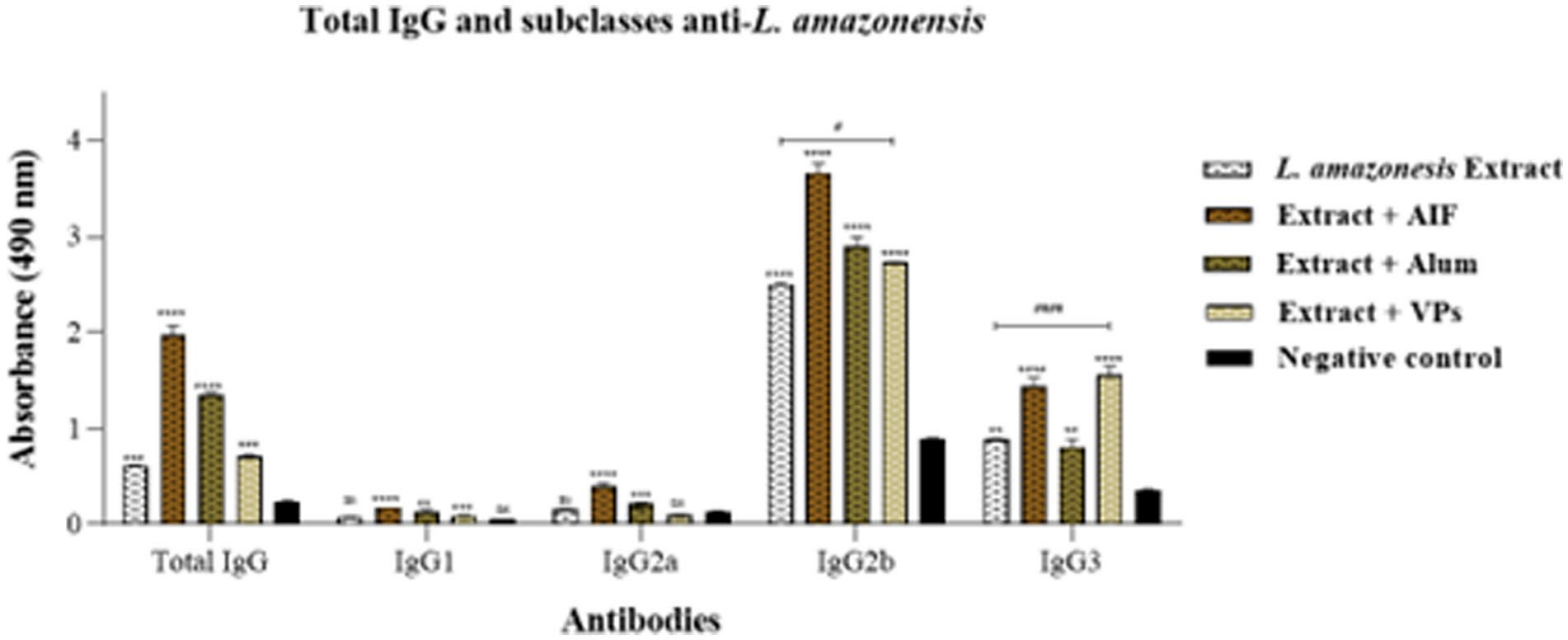
Determination of total IgG anti-*Leishmania* antibodies in animals immunised subcutaneously. The white background bars represent the antibody profile of the group immunised only with the crude protein extract of *L. amazonensis*, whilst the brown bars represent the groups immunised with the crude protein extract in the presence of FIA, alum, and VPs. The profile of non-immunised animals (negative control) is represented by the black bars. The antibody levels are measured against the total crude protein extract of *L. amazonensis*. Each bar represents the mean absorbance obtained from each group of immunised animals and their respective standard deviations 15, 30, 40, and 50 days post-secondary immunisation. Serum samples were analysed at a dilution of 1:25 (v/v). Absorbance at 490 nm is shown on the *Y*-axis. Differences between mean values were considered significant when *p* < 0.05. The symbols “*” and “#” represent the differences observed in the immunised groups compared to the negative control and the difference between the groups immunised only with the crude protein extract vs. crude protein extract mixed with VPs.

Then, the animals were immunised with the second dose of total protein extract of *L. amazonensis* with the different vaccine adjuvants, and serum samples were obtained 15, 30, 40, and 50 days after the secondary immunisation. The anti-*Leishmania* IgG antibody profiles during secondary immunisation are shown in Figure 3. It is observed that after 15 days of secondary immunisation, the total IgG anti-*Leishmania* levels remained more elevated in all immunised groups, when compared to the negative control. The VPs group did not show a significant difference when compared to the group immunised only with the *L. amazonensis* crude protein extract. The FIA group maintained the highest levels amongst all groups, followed by the group immunised with alum.

Additionally, 30 days post-secondary immunisation, the total IgG anti-*Leishmania* levels continued to increase in all immunised groups compared to the negative control. A comparison between the group immunised only with the *L. amazonensis* crude protein extract and the groups immunised with adjuvants (FIA, alum, and VPs) revealed a statistically significant difference. Then, 40 days post-second immunisation, the FIA group presented a higher level compared with negative control, whilst the groups with alum, VPs, and the crude protein extract showed an increase when compared to the negative control (Figure 3). Finally, 50 days post-second immunisation, the FIA group maintained the highest levels, followed by the alum group, the group immunised only with the *L. amazonensis* crude protein extract, and the VPs group. There was no significant difference between the group immunised with the crude protein extract and the VPs group at this time point.

**Figure 4:**
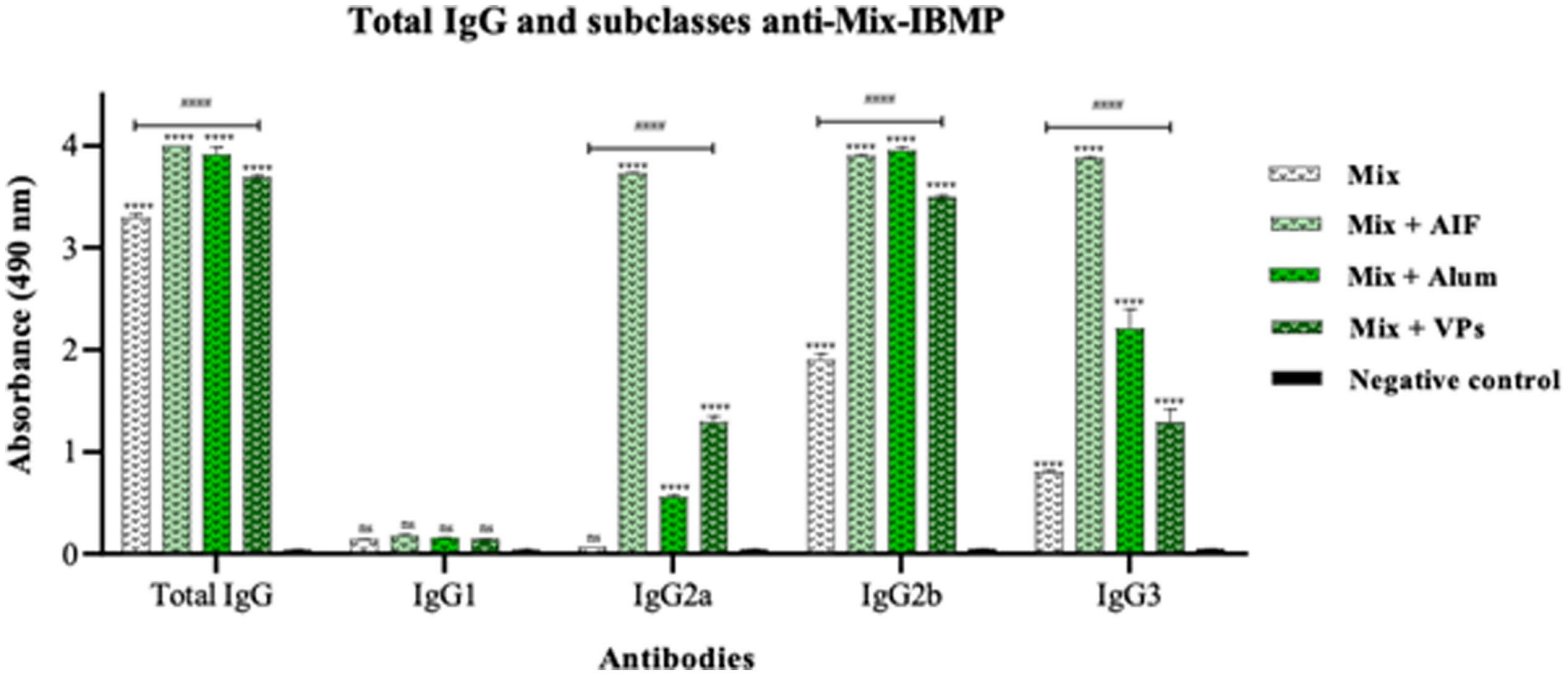
Determination of total IgG antibodies and IgG1, IgG2a, IgG2b, and IgG3 subtypes against *L. amazonensis* in immunised animals 60 days post-immunisation by subcutaneous route. The white background bars represent the antibody profile of the group only immunised with the crude protein extract of *L. amazonensis*, whilst the brown bars represent the groups immunised with the crude protein extract in the presence of FIA, alum, and VPs. The profile of non-immunised animals is represented by the black bars. The antibody levels are measured against the total crude protein extract of *L. amazonensis*. Each bar represents the average absorbance obtained in each group of immunised animals and their respective standard deviations and 60 days post-immunisation. Serum samples were analysed at a dilution of 1:25 (v/v) for total IgG antibodies and 1:10 (v/v) for antibody subtypes. Absorbance at 490 nm is shown on the *Y*-axis. Differences between mean values were considered significant when *p* < 0.05. The symbols “*” and “#” represent the differences observed in the immunised groups compared to the negative control and the difference between the groups immunised only with the crude protein extract vs. crude protein extract together with VPs.

To characterise the nature and quality of the humoral immune response induced during the immunisation process, the IgG antibody subtypes (IgG1, IgG2a, IgG2b, and IgG3) were determined in immunised mice 60 days post-immunisation. The results presented in Figure 4 show that all immunised groups showed statistically significant differences in total IgG anti-*L. amazonensis* compared to the negative control. Then, when comparing the group immunised only with the crude protein extract to the other groups, a significant difference was only found for the FIA and alum groups, with no difference observed in the group immunised with VPs.

In turn, the group immunised with VPs did not show significant anti-*Leishmania* IgG1 antibody levels. In contrast, the groups that received the crude protein extract in combination with FIA or alum presented a significantly higher IgG1 level compared to the negative control and the group immunised only with the crude protein extract (Figure 4). Moreover, the groups immunised with FIA and alum demonstrated statistical significance concerning the anti-*Leishmania* IgG2a antibodies, whilst the VPs group showed no significant differences when compared to the group immunised only with the crude protein extract and the non-immunised group. Interestingly, it was observed that the FIA, alum, and VPs immunised groups showed the presence of anti-*Leishmania* IgG2b antibodies compared to the group that received only the crude protein extract. Additionally, these results indicate that the high anti-*Leishmania* IgG3 antibody levels were measured in the VPs-immunised group, followed by the FIA, alum, and the crude protein extract groups. Then, when comparing the immunised groups, it was observed that the VPs group exhibited a high IgG3 level, with a significant difference when compared to the other groups (FIA, alum, and crude protein extract).

Overall, the analysis of anti-*Leishmania* IgG subtypes suggests that VPs-immunised mice did not induce a significant IgG2a response, unlike the groups with established adjuvants, such as FIA and alum, which exhibited higher levels of these isotypes. However, mice immunised with VPs together with crude protein extraction of *L. amazonensis* demonstrated immunoadjuvant potential, with greater IgG3 production, surpassing the other immunised groups (Figure 4).

### 2.3 Anti-chimeric recombinant antigens antibody profiles in mice immunised with chimeric antigens of *T. cruzi* with different vaccine adjuvants

**Figure 5:**
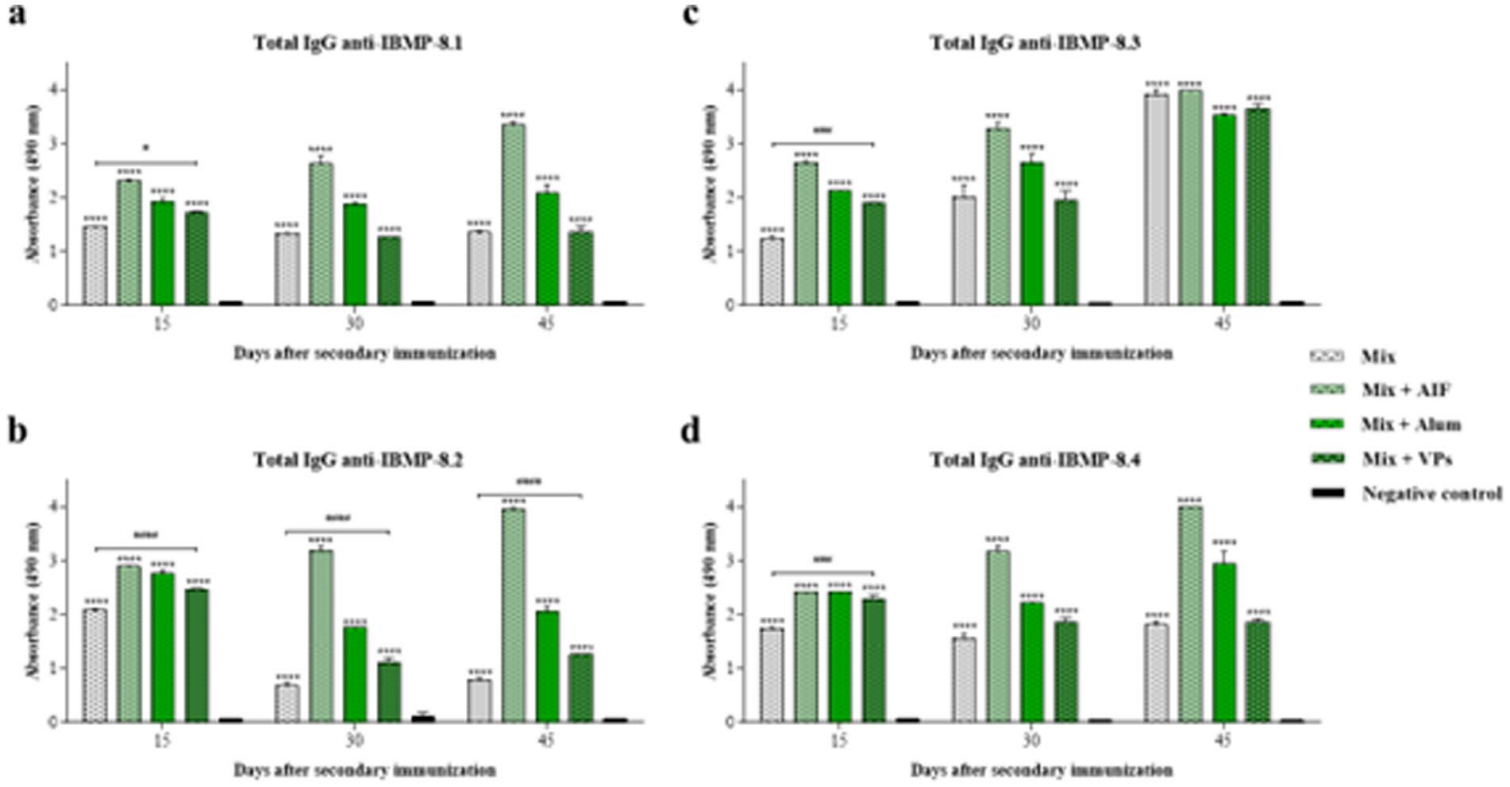
Determination of total IgG anti-IBMPs antibodies in animals immunised by subcutaneous route. The white bars represent the antibody profile of the mix (IBMP-8.1 + IBMP-8.2 + IBMP-8.3 + IBMP-8.4) in the absence of adjuvants, the bars in the green colour scale in the presence of FIA, alum, and VPs, and the profile of non-immunised animals (negative control) is represented by the black bars. The total IgG antibody levels are against each protein anti-IBMP-8.1 **(a)**, anti-IBMP-8.2 **(b)**, anti-IBMP-8.3 **(c)**, and anti-IBMP-8.4 **(d)**. Each bar represents the mean absorbances obtained in each group of immunised animals and their respective standard deviations 15, 30, and 45 days post-immunisation. Serum samples were analysed at a dilution of 1:100 (v/v). Absorbance at 490 nm is shown on the *Y*-axis. Differences between mean values were considered significant when *p* < 0.05. The “*” and “#” signs represent the differences observed in the immunised groups compared to the negative control and the difference between the mix with VPs vs. ix immunised groups, respectively. The acronym “ns” stands for statistical non-significance.

First, sera from immunised mice 15, 30, and 45 days post-immunisation were used to quantify specific antibodies against chimeric recombinant proteins of *T. cruzi* by ELISA, in order to evaluate the immunisation process with chimeric recombinant proteins (denominated here IBMP) of *T. cruzi,* formulated with different vaccine adjuvants (alum, FIA, and VPs). A mixture (Mix) of four recombinant chimeric proteins (IBMP-8.1, IBMP-8.2, IBMP-8.3, and IBMP-8.4) mixed with different vaccine adjuvants was used for immunisation. Figure 5 represents the IgG antibody profile against each recombinant chimeric protein of *T. cruzi*. The total IgG antibody profiles were against each IBMP antigen: anti-IBMP-8.1 (a), anti-IBMP-8.2 (b), anti-IBMP-8.3 (c) and anti-IBMP-8.4 (d).

The results show (Figure 5) that the groups immunised with adjuvants during the primary immunisation process present statistical disparities when compared to the negative control. The group with only the Mix presents different total IgG antibody profile for each IBMP protein. The anti-IBMP-8.1 (Figure 5a) and anti-IBMP-8.2 (Figure 5b) antibody profiles are higher than the negative control, whilst anti-IBMP-8.3 (Figure 5c) and anti-IBMP-8.4 (Figure 5d) are even more elevated. However, they did not present statistically significant differences at any of the times analysed, or in relation to the negative control.

In this scenario, the association of Mix with FIA showed a difference in total IgG profile when compared to the negative control, with a higher antibody profile observed by inducing the IBMP-8.2 (Figure 5b), IBMP-8.3 (Figure 5c) and IBMP-8.4 proteins (Figure 5d). Only anti-IBMP-8.1 antibodies (Figure 5a) presented lower antibody profiles when compared to the induction of the others; even so, this is the group with the highest antibody profile at all times and proteins in the first dose (Figure 5).

As shown in Figure 5, the immunoadjuvant potential of VPs (VP1, VP2 and VP3) for the induction of total IgG antibodies was evaluated in association with the Mix (Mix + VPs), demonstrating no statistical difference against the IBMP-8.1 (Figure 5a) and IBMP-8.4 proteins (Figure 5d) compared to the negative control. There was a difference in anti-IBMP-8.2 (Figure 5b) in the three analysed times points and anti-IBMP-8.3 (Figure 5c) only for the 15-day time point.

The statistical differences between the groups at the three time points were analysed and it was observed that there was a statistically significant difference in the antibody profiles at 15 days between Mix vs. Mix with FIA; Mix vs. Mix with alum; Mix with FIA vs. Mix with alum; and Mix with FIA vs. Mix with VPs in relation to the IBMP-8.1 protein (Figure 5a). For IBMP-8.2 (Figure 5b), in addition to the difference between Mix vs. Mix with VPs (demonstrated in the graph by the “#” sign), there were differences between: Mix vs. Mix with FIA; Mix with FIA vs. Mix with alum; Mix with FIA vs. Mix with VPs; and Mix with alum vs. Mix with VPs. Taken together, regarding the IBMP-8.3 protein (Figure 5c), there was only no difference between Mix vs. Mix with VPs, and the same occurred for the profile related to IBMP-8.4 (Figure 5d).

In turn, there was no significant difference at 30 days post-immunisation for anti-IBMP-8.1 antibodies (Figure 5a) between the immunised groups Mix vs. Mix with alum; Mix vs. Mix with VPs; and Mix with alum vs. Mix with VPs. Regarding anti-IBMP-8.2 antibodies (Figure 5b), there was no difference when comparing Mix with alum vs. Mix with VPs. In the antibody profiles for the anti-IBMP-8.3 protein (Figure 5c), there was a difference between the groups: Mix vs. Mix with FIA; Mix vs. Mix with alum; Mix with FIA vs. Mix with alum; Mix with FIA vs. Mix with VPs; and Mix with alum vs. Mix with VPs. On the other hand, in the antibody profiles against IBMP-8.4 (Figure 5d), there were differences between: Mix vs. Mix with FIA; Mix with FIA vs. Mix with alum and Mix with FIA vs. Mix with VPs.

In relation to the anti-IBMP-8.1 antibodies (Figure 5a) at 45 days post-immunisation, a difference was only observed between: Mix vs. Mix with FIA; Mix with FIA vs. Mix with alum and Mix with FIA vs. Mix with VPs. However, the scenario changes for anti-IBMP-8.2 antibodies (Figure 5b), presenting a difference only in the comparison of Mix vs. Mix with alum and Mix with alum vs. Mix with VPs. Furthermore, a difference is noted for anti-IBMP-8.3 antibodies (Figure 5c) between: Mix vs. Mix with FIA; Mix vs. Mix with alum; Mix with FIA vs. Mix with alum; and Mix with FIA vs. Mix with VPs. Then, in the analysis related to IBMP-8.4 antibodies (Figure 5d), no difference is observed when comparing Mix vs. Mix with alum; Mix vs. Mix with VPs and Mix with alum vs. Mix with VPs.

**Figure 6:**
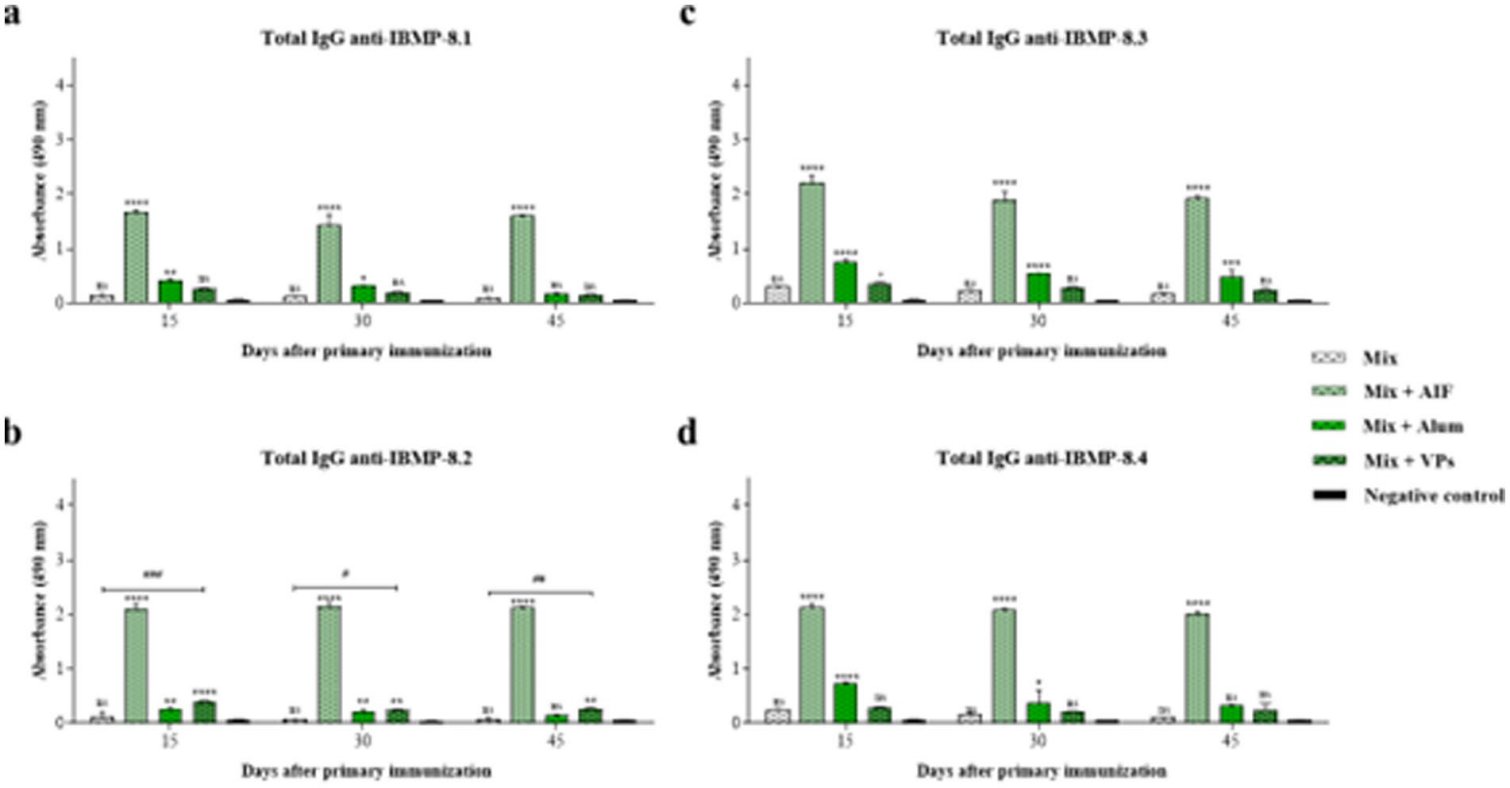
Determination of total IgG anti-IBMPs antibodies in mice immunised subcutaneously after secondary immunisation. The white bars represent the antibody profiles of the mix (IBMP-8.1 + IBMP-8.2 + IBMP-8.3 + IBMP-8.4) in the absence of adjuvants, the bars in the green colour scale in the presence of FIA, alum, and VPs, and the profile of non-immunised mice (negative control) is represented by the black bar. The antibody levels are against each protein anti-IBMP-8.1 **(a)**, anti-IBMP-8.2 **(b)**, anti-IBMP-8.3 **(c)**, and anti-IBMP-8.4 **(d)**. Each bar represents the mean absorbances obtained in each group of immunised mice and their respective standard deviations 15, 30, and 45 days post-immunisation. Serum samples were analysed at a dilution of 1:100 (v/v). Absorbance at 490 nm is shown on the *Y*-axis. Differences between mean values were considered significant when *p* < 0.05. The “*” and “#” signs represent the differences observed in the immunised groups compared to the negative control and the difference between the mix with VPs vs. mix immunised groups, respectively. The acronym “ns” stands for statistical non-significance.

Secondary immunisation of the mice was performed after characterising the antibody response of mice immunised with a single dose (Figure 5). In this scenario, the results presented in Figure 6 show that the group immunised with Mix administered without adjuvants shows a considerable increase in total IgG profiles for all proteins and time points after secondary immunisation. Notably, at 45 days post-immunisation, the anti-IBMP-8.3 antibody (Figure 6c) levels increase with time, whilst the anti-IBMP-8.1 (Figure 6a) and anti-IBMP-8.4 (Figure 6d) profiles remain the same. Only the profile of antibodies induced by IBMP-8.2 protein (Figure 6b) decrease at 30 and 45 days.

Immunised mice with Mix with alum showed a significant change for total IgG anti-IBMPs antibodies in the second dose and statistically differs from the first immunisation. The Mix in association with FIA continued to be the group that demonstrated the highest total IgG profile at all times and proteins evaluated. The antibody profiles induced by all proteins increased over time, reaching their highest levels at 45 days. In addition, the association of Mix with VPs, at all times and all immunisations evaluated, presented significant differences compared to the negative control, with an increase in total IgG induced by the IBMP-8.3 protein (Figure 6c), reaching its highest level at 45 days. The lowest antibody profile is reported for anti-IBMP-8.2 antibodies (Figure 6b) at 30 and 45 days, whilst the anti-IBMP-8.1 antibody (Figure 6a) and anti-IBMP-8.4 antibody (Figure 6d) profiles remained similar at all times evaluated.

The statistical differences between the immunised groups at the three time points were also analysed and it was observed that no difference was found for the following immunised groups at 15 days post-immunisation: between Mix with alum vs. Mix with VPs for the anti-IBMP-8.1 antibodies (Figure 6a); between Mix with FIA vs. Mix with alum for anti-IBMP-8.2 antibodies (Figure 6b); between Mix with alum vs. Mix with VPs for the anti-IBMP-8.3 antibodies (Figure 6c); and for anti-IBMP-8.4 antibodies (Figure 6d) between the groups Mix with FIA vs. Mix with alum as presented by anti-IBMP-8.2 antibodies; Mix with FIA vs. Mix with VPs; and Mix with alum vs. Mix with VPs for anti-IBMP-8.1 antibodies.

Next, there was no statistically significant difference for the antibody profiles against IBMP-8.1 (Figure 6a), IBMP-8.3 (Figure 6c) and IBMP-8.4 (Figure 6d) proteins at 30 days post-immunisation between Mix vs. Mix with VPs. However, a difference is observed between all groups for the antibody profiles against the IBMP-8.2 protein (Figure 6b). In turn, no statistically significant difference was found at 45 days post-immunisation for anti-IBMP-8.1 antibodies (Figure 6a) or anti-IBMP-8.4 antibodies (Figure 6d) between Mix vs. Mix with VPs, as well as at 30 days. A difference is observed between all groups for anti-IBMP-8.2 antibodies (Figure 6b) at 45 days post-immunisation, as well as at 30 days. Lastly, a difference is observed only between Mix vs. Mix with alum for anti-IBMP-8.3 antibodies (Figure 6c).

**Figure 7:**
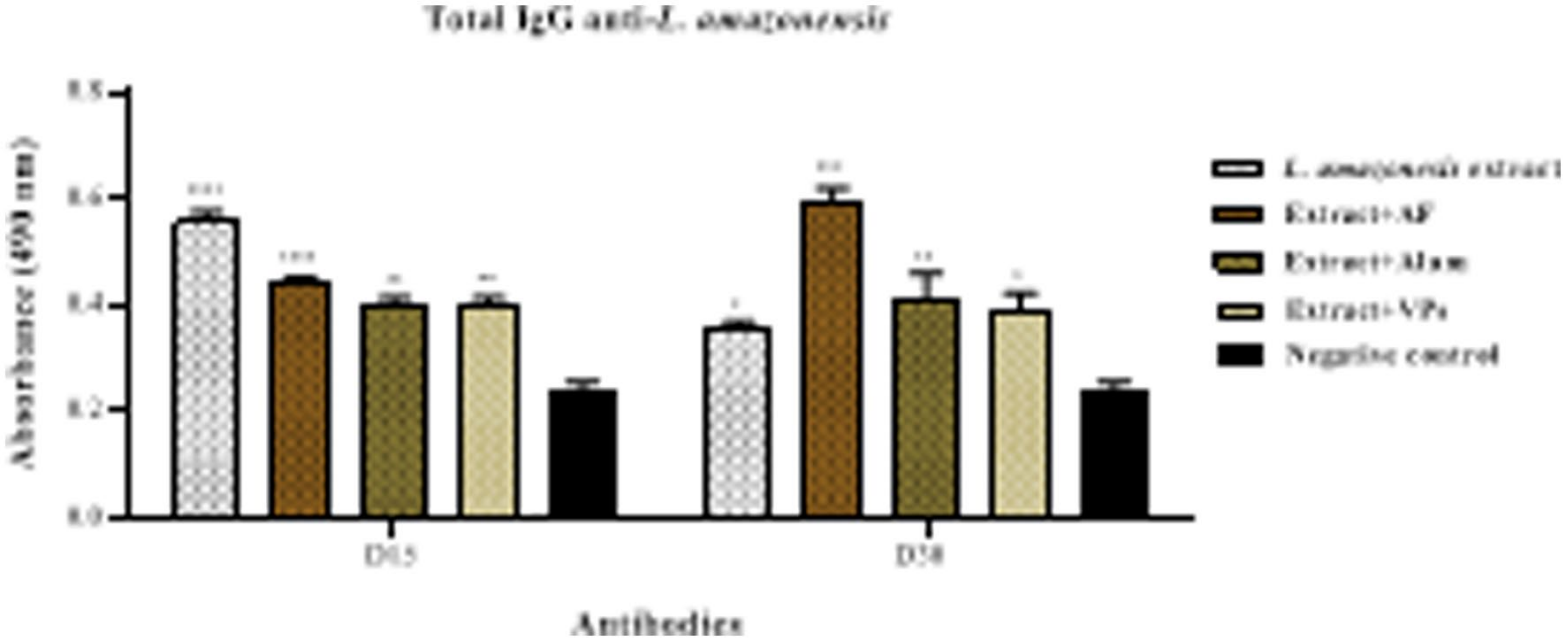
Determination of total IgG and subclasses IgG1, IgG2a, IgG2b, and IgG3 anti-mix-IBMP antibodies in mice immunised subcutaneously. The bars in white background represent the antibody profile of mix (IBMP-8.1 + IBMP-8.2 + IBMP-8.3 + IBMP-8.4 in antigenic mixture) in the absence of adjuvants, bars on the green colour scale in the presence of FIA, alum, and VPs, and the antibody profiles of non-immunised mice (negative control) are represented by the black bars. Each bar represents the mean absorbances obtained in each group of immunised animals and their respective standard deviations, after 70 days post-booster. Serum samples were analysed at 1:50 dilution (v/v). Absorbance at 490 nm is shown on *Y*-axis. Differences between mean values were considered significant when *p* < 0.05. Signs “*” and “#” represent differences observed in the immunised groups compared to the negative control and difference between the immunised groups mix with VPs vs. mix, respectively. The acronym “ns” stands for statistical non-significance.

Then, in order to characterise the nature and quality of the humoral immune response induced during the immunisation process with chimeric recombinant proteins of *T. cruzi*, the IgG antibody subtypes (IgG1, IgG2a, IgG2b, and IgG3) were determined in the immunised mice at 70 days post-second immunisation (Figure 7). Considering that we already know the total IgG profile against each protein separately, at this stage it was decided to present the profile against the Mix of the four proteins in antigenic mixture (Mix-IBMP) to provide a new perspective of the profile and a new approach to interpreting the results.

Therefore, the data presented in Figure 7 show the profiles of the different isotypes of IgG antibodies against chimeric recombinant protein of *T. cruzi* associated with different adjuvants. The immunised group associated with FIA continues to exhibit the highest IgG profile when compared to the negative control. There is no induction of IgG1, but it is the group that induces the most IgG2a and IgG3, whilst the profile for IgG2b is similar to that of Mix associated with alum. In addition, the total IgG profile for Mix with alum is similar to that observed when Mix was associated with FIA, with 97-times higher levels than the non-immunised mice. Moreover, there was no induction of IgG1, there was little induction of IgG2a with a lower profile compared to the association with FIA and VPs, IgG2b was equal to the association with FIA and IgG3 had a lower profile, but still higher than that observed with VPs. In this context of IgG subtypes, the results demonstrated that there was no induction of IgG1, whilst the IgG2a and IgG3 antibodies induction displayed similar profiles, and IgG2b showed higher levels of antibodies produced. All antibodies produced presented higher profiles than those observed when Mix was administered alone (Mix), mainly IgG2b. VPs was able to induce higher IgG2a levels in relation to alum.

Furthermore, all antibody subclasses produced 70 days after the second dose in association with VPs had a better profile than when Mix was administered alone, especially IgG2b. Mix, whether associated or not with adjuvants, did not produce IgG1 even 70 days after the second dose, including with VPs. After the second dose, Mix alone was capable of inducing levels of total IgG and subclasses at all times and for all proteins, including at 70 days after the second dose. No group showed a statistically significant difference in the IgG1 profile. The highest IgG levels in the second dose were induced by the IBMP-8.2 and IBMP-8.3 proteins. The IgG2b subclass was the most produced in all groups compared to 70 days after the second dose.

## 3 Discussion

In this study, we showed that native antigens from *L. amazonensis* exhibited similar antibody response profiles when administered in a murine model in combination with FIA, alum, and VPs, with no significant differences between them or compared to non-immunised mice in the experiment, as evaluated at 15 and 30 days after the first dose. This response may be attributed to the adjuvant properties, which initially hinder processing of the antigen by antigen-presenting cells, prolonging its release and consequently modulating the immune response. This modulation may have contributed to the smaller difference observed amongst the groups treated with adjuvants and VPs by promoting a more gradual and controlled response (Zhao et al., 2023b; Powers et al., 2007) compared with *L. amazonensis* crude protein extract. IgG antibody profiles were elevated after 30 days in all groups, with particular emphasis on those administered FIA, which exhibited the highest levels (*p* < 0.05). The observed immunological profile is widely recognised in the scientific literature. FIA is known for its ability to stimulate a robust immune response, primarily inducing a Th1-type cellular response (Cargnelutti et al., 2016).

A difference was observed 30 days after the booster dose and comparing the group immunised only with the total *L. amazonensis* crude protein extract to the groups immunised with adjuvants (FIA, alum, and VPs, suggesting modulation of the immune response by the presence of these components in the formulation. In the context of the VPs group, this significance at this collection point may indicate an immunoadjuvant action of the VPs, favouring induction of a more prolonged and effective immune response over time compared to the isolated crude protein extract. In this context, Cargnelutti et al. (2016) implemented an immunisation protocol using vaccine preparations with total *L. amazonensis* crude protein extract with adjuvants, performed the first immunisation, and administered a booster dose 3 weeks later, with collections conducted at 21 and 36 days after the first immunisation (Yasmin et al., 2022). An ELISA was executed to characterise the humoral response against *L. amazonensis* in this process. In parallel to our data, the results obtained showed a significant increase in total IgG profile against *L. amazonensis* after the booster dose. The response profile of the groups that received only the crude protein extract of LaPSA-VLPs in both studies was also similar.

Still regarding the immunisation protocol with native antigens, the analysis of the IgG anti-*L. amazonensis* subclasses performed 60 days after the second dose included IgG1, IgG2a, IgG2b, and IgG3. As shown in Figure 4, the group immunised with VPs did not exhibit significant IgG1 levels. In contrast, the groups that received the *L. amazonensis* crude protein extract in combination with established adjuvants in the literature (FIA and alum) showed significantly higher IgG1 profiles compared to the negative control and the group immunised only with the crude protein extract.

Infection by *Leishmania* leads to induction of a complex immune response, characterised by both cell-mediated reactions and antibody production (Conde et al., 2022). The nature of the cellular immune response plays a crucial role in determining the humoral immune response by inducing production of specific antibody isotypes (Kotb et al., 2021). A detailed characterisation of immunoglobulin subclasses, such as IgG1, IgG2a, IgG2b, and IgG3, are fundamental for understanding the polarisation of the immune response and the type of dominant immunological profile (Th1 or Th2). This analysis can indicate whether the organism is developing a protective response or susceptibility to the disease (Dubie and Mohammed, 2020).

In leishmaniasis, T cell subpopulations are presented through a cure-disease paradigm via the Th1/Th2 relationship. The Th1-mediated response is related to mechanisms of parasite control, whilst the Th2 response profile is linked to susceptibility to the infection (Akbari et al., 2022; Vakili et al., 2020). Cytokines derived from the Th1 profile are thought to stimulate secretion of IgG immunoglobulins by B lymphocytes. Conversely, when the response profile is Th2, B lymphocytes are stimulated to secrete IgE and IgA antibodies, and subclasses of IgG, stimulating an inflammatory response mediated by eosinophils, basophils, and mast cells (Coffman et al., 1988). Thus, the relative production of these isotypes can serve as a marker for inducing Th1 and Th2 immune responses (Dubie and Mohammed, 2020).

Studies by Coffman et al. (1988) and Visciano et al. (2012) reinforce the idea that the IgG pattern and its subclasses plays a crucial role in the course of infections, with studies suggesting that IgG1 is an indicator of the Th2 response in the case of infection caused by *L. amazonensis*, directly related to disease susceptibility (Coffman et al., 1988; Visciano et al., 2012). In a study conducted by Marlais et al. (2018), it is suggested that the prevalence of the IgG1 subtype can be used as a biomarker for disease relapse post-chemotherapy treatment for VL, as it has been demonstrated that levels of this immunoglobulin significantly decreased in cases cured after treatment, whilst this immunoglobulin appeared at elevated concentrations when the disease progressed in the patients recruited in the study (Marlais et al., 2018; Salih et al., 2025). However, it must be considered that variables can influence the course of the establishment or control of the disease.

The literature documents that the IgG2a isotype is related to polarisation of the Th1 response, a response profile linked to the control of the parasite in mouse models (Rostamian et al., 2017). When analysing Figure 3 related to 60 days after the booster dose, it was evident that the VPs group did not present significant values for IgG2a compared to the negative control. However, for further study, it will be necessary to evaluate the antibody isotype profile in relation to cytokine production as measured by Th1 and Th2 lymphocytes. This includes the potential ability of this immunisation process to protect animals experimentally infected with *L. amazonensis*.

A study conducted by Cargnelutti et al. (2016), in which BALB/c animals were subjected to first-generation vaccine preparations consisting of *L. amazonensis* crude protein extract with adjuvants, it was inferred that the mice in the group presenting a high IgG2a/IgG1 ratio were protected when subjected to an experimental challenge with *L. amazonensis* (Cargnelutti et al., 2016). Conversely, there was no protection in the group where the IgG2a/IgG1 ratio was low against the challenge. The studies by Rostamian et al. (2017) and Ebrahimpoor et al. (2013) are also in agreement with the results of Cargnelutti, although different strains were used in both studies (Rostamian et al., 2017; Ebrahimpoor et al., 2013).

Despite such findings, it is important to emphasise that variables such as the strain used in the infection, the species undergoing the infectious process, and the inoculation site are extremely important variables for determining the course of the disease/cure; however, elucidation of such variables is still not fully established, making it crucial to develop studies that seek to characterise the humoral and cellular immune response to clarify such processes.

Regarding the IgG2b profile 60 days after the booster dose, it was observed that the FIA, VPs, and alum groups exhibited 1.4-, 1.1-, and 1.2-fold higher IgG2b levels than the group which only received the *L. amazonensis* crude protein extract, suggesting an enhanced immune response due to the presence of these components in the vaccine preparations.

It is important to point out that the profile of this immunoglobulin is still not fully clarified in animal models immunised with the *L. amazonensis* strain, highlighting the need for research to characterise this profile to elucidate whether this immunoglobulin is a marker of the Th1 or Th2 profile. In the study by Mohrs et al. (1999), BALB/c mice challenged with *L. major* demonstrated that the group with the highest protection levels against acute infection showed higher titters of IgG2a, IgG2b, and IgG3 compared to IgG1 and IgG3 levels in the same animals, initially modulating a Th1 response (Mohrs et al., 1999).

The comparative analysis amongst the groups in this study revealed that the group treated with VPs exhibited the highest IgG3 profiles, with a significant difference compared to the other groups (FIA, alum, and the group treated with the crude protein extract). These results indicate a potentiated immune response in the groups that received VPs, FIA, and alum, respectively. Overall, this isotype predominated in the VP group at all collection points established for the analysis.

Although there is a scarcity of studies on IgG3 profiles in *L. amazonensis* infections in murine models, a study by de Souza et al. (2005) evaluated IgG subclasses in the serum of 37 patients diagnosed with ACL using indirect ELISA with *L. amazonensis* antigens, and identified a predominance of IgG subclasses in the following order: IgG1 > IgG3 > IgG2 > IgG4 in cases of cutaneous and mucocutaneous leishmaniasis. The predominance of IgG1, IgG2, and IgG3 isotypes was associated with a Th1 immune response profile, characterised by the mechanism of resistance to the parasite (Casella and Mitchell, 2008). However, it is important to highlight that the reproducibility of data between murine models and human studies is limited due to the intervening factors already mentioned in this work.

Despite the scarcity of information about IgG3 and its role in *Leishmania* infection, the literature suggests that this isotype plays an important role in the opsonisation and destruction of cells infected by pathogens rather than merely neutralising them. Additionally, IgG3 may contribute to generating cytotoxic CD8+ T cell responses, which directly eliminate infected host cells (Casella and Mitchell, 2008). These characteristics suggest an immunological advantage in combating *Leishmania*. However, further investigations are needed to clarify the exact role of this antibody in the context of *Leishmania* infection.

A study by Queiroz et al. (2021a) used *Triatoma virus* VLPs combined with recombinant chimeric antigens of *T. cruzi* in BALB/c mice, observing that the highest total IgG profiles were for VP3. However, when comparing these results to those obtained in the present study, a disparity was noted, as VP1 presented the highest total IgG profiles herein. These findings suggest that when the proteins are associated with the *L. amazonensis* crude protein extract, the immune response is modulated differently compared to their isolated administration.

The applicability of the VLP and/or VPs platform in new vaccine formulations is on the rise, constituting an appropriate approach given that they are particles with high immunogenicity, providing a promising possibility for novel immunisation protocols as they are capable of inducing a protective immune response with a safety profile (Chroboczek et al., 2014; Facciolà et al., 2019).

Another important point is that the antibody profiles after the first formulation dose associated with VPs decreases over time, suggesting the need for a booster dose. This decrease may be due to the natural degradation of antibodies or decreased immune memory. Boosting helps revive this response by stimulating production of new antibodies and activating memory cells (Seder and Hill, 2000; Zimmermann and Curtis, 2019).

The switch from antibodies to IgG1 is triggered by IL-4, a cytokine involved in the Th2 response, whilst IgG2a is induced by IFN-*γ*, a cytokine involved in Th1. As mentioned previously, a prophylactic response that also induces Th1 is ideal for *T. cruzi* infection. In the context of Chagas disease, only a prophylactic Th2 response can exacerbate the mechanisms of infection by *T. cruzi*, as Th2 cells block those involved in the Th1 response, potentially allowing the protozoan to continue in the organism. The cellular immune response of the Th1 type is also desired to act against maintenance of the parasite, especially the intracellular, replicative forms involved in the progression to chronic Chagas disease (Fakharzadeh et al., 2013; Javadi et al., 2022).

The IgG2b and IgG3 subtypes produced at higher levels in the two protocols proposed in this study are essential, since in mice they present cytophilic characteristics which favour increased phagocytosis through opsonisation and activate the complement system. Finally, the need for a booster dose in both protocols ensured the maintenance and improvement of the immune response over time, as the primary response may not be sufficient to provide complete protection against infection or maintain adequate levels of antibodies (Morillo et al., 2015; Plotkin, 2010; Amanna and Slifka, 2011; Husain et al., 2011). Additionally, its important to mention that this study represents a first step in characterising potential new vaccine adjuvants in the context of Chagas disease and leishmaniasis. Therefore, it has some limitations, mainly because it characterises the immunisation process exclusively by the humoral response and does not take into account the activation process of CD4+ T lymphocytes (Th1 and Th2, for example) and CD8+ T cells.

In summary, this study represents a pioneering advance in the use of recombinant chimeric antigens of *T. cruzi* and total antigens of *L. amazonensis*, both associated with promising vaccine adjuvants, which should be evaluated more precisely in a next stage of immunisation, taking into account the different (i) routes of antigen administration; (ii) the nature and dose of antigens administered; (iii) the activation process of CD4+ and CD8+ T lymphocytes in cytokine production; and (iv) proof-of-concept aspects regarding infection control in the context of Chagas disease and leishmaniasis.

## 4 Materials and methods

### 4.1 Obtaining recombinant structural proteins from *Triatoma virus*

The genes of the VPs VP1, VP2 and VP3 of *Triatoma virus* (TrV) were cloned into pET3d (+), expressed in *E. coli* BL21 (DE3) and induced with IPTG. The recombinant proteins were purified by size exclusion chromatography and characterised by SDS-PAGE and immunoblotting using rabbit polyclonal serum anti-VP1, anti-VP2, and anti-VP3 antibodies.

### 4.2 Obtaining promastigote forms of *L. amazonensis* and preparation of crude protein extract

*L. amazonensis* strain was kindly provided by the Institute of Hygiene and Tropical Medicine at the Nova University of Lisbon, Portugal. Promastigote forms of *L. amazonensis* were cultured for 7 days in RPMI-1640 medium (Roswell Park Memorial Institute 1,640), supplemented with 10% (v/v) inactivated Foetal Bovine Serum (FBS) and 2% (v/v) streptomycin/penicillin antibiotics (100 IU/mL). The cultures were maintained at 27 °C in a BOD incubator (ASP, SP-500) (Limoncu et al., 1997). Maintenance was performed every 2-3 days due to changes in the pH of the medium, detected by the colour change from pink to yellow. After reaching the stationary growth phase with a concentration of 1 × 10^6^ parasites/mL in the *Leishmania* spp., the culture was centrifuged at 3000 × g and 4 °C for 10 min. The obtained pellets were washed three times with sterile Phosphate-Buffered Saline (PBS), then centrifuged at 3000 × g and 4 °C for 10 min each wash. Pellets containing the parasite were subsequently resuspended in 1 mL of PBS with protease inhibitor, pH 7.0. The content was subjected to six freeze-thaw cycles, followed by mechanical agitation.

### 4.3 Obtaining chimeric recombinant antigens of *T. cruzi*

*T. cruzi* recombinant proteins, called IBMP-8.1, IBMP-8.2, IBMP-8.3 and IBMP-8.4, were produced at the Instituto Carlos Chagas (Oswaldo Cruz Foundation, FIOCRUZ, Paraná, Brazil) by synthetic genes optimised for expression in *E. coli* (Gen-Script, United States). After subcloning into pET28a, the recombinant proteins were purified using affinity chromatography via a 6xHis tag. These recombinant proteins are protected by a patent application at Fiocruz-Brazil (No. 1020180161407) (Santos et al., 2018).

### 4.4 Immunisations in animal models

In this work, two immunisation models were studied: the first strategy involved native *L. amazonensis* antigens and the immunisation schedule is shown in Table 1. The second strategy involved chimeric recombinant antigens of *T. cruzi* and the immunisation protocol is shown in Table 2. All antigens and proteins were eluted in PBS (pH 7.2) and mixed with each of the adjuvants in a 1:1 (v/v) ratio using a vortex.

**Table 1:** Immunisation groups in mouse models (protocol with native antigens)^a^.

Experimental groups	First immunisation	Second immunisation
Group 1: *L. amazonensis* crude protein extract	100 μg of crude protein extract: groups 1, 2, 3 and 4; and 33.33 μg of each VPs: group 4.	The crude protein extract and VPs doses were 50 μg.
Group 2: crude protein extract + FIA		
Group 3: crude protein extract + alum		
Group 4: crude protein extract + VPs		
Group 5: negative control		

a *n* = 5 per group. Crude protein extract: promastigote forms of *L. amazonensis*; FIA, Freund’s Incomplete Adjuvant; alum, Aluminium hydroxide; VPs, Mix of VP1, VP2, and VP3. Group 5: PBS.

**Table 2:** Immunisation groups in mouse models (protocol with chimeric recombinant antigens)^a^.

Experimental groups	First immunisation	Second immunisation
Group 1: *T. cruzi* chimeric recombinant antigens (mix)	12.5 μg of each IBMP, mix with 50 μg in groups 1, 2, 3 and 4; 16.66 μg of each VP, VPs with 49.98 μg in group 4.	Occurred with the same dose on day 45 after the first dose.
Group 2: mix + FIA		
Group 3: mix + alum		
Group 4: mix + VPs		
Group 5: negative control		

a *n* = 3 per group. Mix of IBMP-8.1, IBMP-8.2, IBMP-8.3, and IBMP-8.4; FIA, Freund’s Incomplete Adjuvant; alum, Aluminium hydroxide; VPs, Mix of VP1, VP2, and VP3. Group 5: PBS.

All procedures involving animals were conducted in accordance with the guidelines 80 of the National Council for the Control of Animal Experimentation (CONCEA) and international standards of animal welfare. The study was approved by the Animal Ethics Committee of the Federal University of Rio Grande do Norte (CEUA/UFRN), protocols numbers 080/2017 and 041/2023). Female BALB/c mice (20–30 g, 8 weeks old) were maintained under controlled lighting (12 h light/dark) and temperature (22 °C) conditions. The mice were obtained from the Animal Facility of the Health Sciences Centre of the Federal University of Rio Grande do Norte, Brazil. Immunisations were administered subcutaneously using a syringe (Omnican^®^ 100). After the interventions, the mice received an overdose of xylazine (Syntec, São Paulo, Brazil) and ketamine (Vetnil, São Paulo, Brazil) intraperitoneally, followed by euthanasia by cardiac exsanguination.

### 4.5 Obtaining sera from immunised mice and determination of anti-*L. amazonensis* antibodies or anti-recombinant *T. cruzi* proteins

Serum samples were initially collected after whole blood clotting by terminal excision of the tail or by cardiac exsanguination after of the euthanasia to determine specific antibodies from immunised mice. Then, samples were maintained at 37 °C for 30 min and then at 5 °C for 1 h. Serum was subsequently obtained by centrifugation at 5,000 rpm for 10 min. The serum samples were fractionated and stored at −20 °C for screening murine antibodies specific anti-*L. amazonensis*, anti-recombinant *T. cruzi* proteins (IBMPs proteins), and anti-structural proteins from VP1, VP2, and VP3 by Enzyme Linked Immunosorbent Assay (ELISA).

Next, 96-well microplates (Nunc-Immuno MicroWell, United States) were sensitised with 0.1 M bicarbonate buffer (pH 8.5) with 100 ng/well of *L. amazonensis* crude protein extract or 50 ng/well of IBMP recombinant proteins of *T. cruzi*. The plates were sensitised overnight at 5 °C for both protocols (Queiroz et al., 2021a). The microplates were subsequently washed three times with PBS containing 0.05% Tween20 (Promega, United States). Then, the plates were blocked for 1 h at room temperature (25 °C) with 1% (w/v) skim milk in PBS. After five additional washes, sera from immunised mice were added in serial dilutions: 1:25 for total IgG and 1:10 for IgG subtypes (for determining anti-*L. amazonensis* antibodies), and 1:100 or 1:50 for total IgG and 1:50 for IgG subtypes (for determining anti-recombinant *T. cruzi* proteins), all in antibody dilution buffer. After five more washes, HRP-conjugated antibodies (rabbit anti-mouse IgG diluted 1:4.000-Sigma-Aldrich, United States; rabbit anti-mouse IgG1 diluted 1:2.000-Caltag Laboratories, United States; rabbit anti-mouse IgG2a diluted 1:2.000-Caltag Laboratories, United States; rabbit anti-mouse IgG2b diluted 1:2.000-Invitrogen, United States, and rabbit anti-mouse IgG3 diluted 1:2.000-Sigma-Aldrich, United States) were added in antibody buffer and incubated for 1 h at room temperature. Subsequent washes were performed, and the plates were incubated for 30 min at room temperature with a substrate solution consisting of 10 mL of citrate buffer (pH 5.0) with 10 mg of o-phenylenediamine dihydrochloride (OPD: Sigma-Aldrich, United States) and 10 μL of hydrogen peroxide 30%. All sera from immunised mice were analysed in duplicate, and the final reaction absorbance at 490 nm was measured using a spectrophotometer (Epoch, BioTek Instruments, Winooski, VT, United States).

### 4.6 Statistical analysis

Data relating to the differences between the means of the immunised groups and the non-immunised control groups during primary and secondary immunisations, days after immunisations, antigens and antibody profiles were analysed according to Analysis of Variance (ANOVA), followed by the Tukey’s multiple comparison post-test by GraphPad Prism© software version 8.4 (GraphPad Inc., Illinois, Chicago, United States). The significance of differences between means was established when *p* < 0.05.

## 5 Conclusion

In conclusion, the study showed that immunisation protocols based on native antigens from *L. amazonensis* combined with different adjuvants, including the VPs of the *Triatoma virus*, were able to induce total IgG antibodies against *L. amazonensis*. The group immunised with VPs exhibited a significant increase in IgG2b and IgG3 subtypes, indicating a possible modulated Th1 immune response. Additionally, the antigen mixture (mix), either alone or combined with adjuvants, demonstrated the ability to induce antibody subclasses such as IgG2a, IgG2b, and IgG3, despite not stimulating IgG1 production, highlighting its potential in intracellular infections, such as those caused by *T. cruzi*, the etiological agent of Chagas disease. In summary, the immunoadjuvant property of VPs was confirmed through induction of IgG2a and IgG3 antibodies in immunised mice, reinforcing their role as a promising tool in vaccine strategies. These findings indicate that the VPs may contribute to a differentiated modulation of the immune response, highlighting the importance of future studies to explore their role as a vaccine adjuvant. However, further immunogenicity and proof-of-concept studies are needed to characterise VPs as potential vaccine adjuvants.

## Data Availability

The raw data supporting the conclusions of this article will be made available by the authors, without undue reservation.

## References

[ref1] Akbari M. Oryan A. Hatam G. (2022). Immunotherapy in treatment of leishmaniasis. Immunol. Lett. 233, 80–86. doi: 10.1016/j.imlet.2021.03.011, 33771555

[ref2] Amanna I. J. Slifka M. K. (2011). Contributions of humoral and cellular immunity to vaccine-induced protection in humans. Virology 411, 206–215. doi: 10.1016/j.virol.2010.12.016, 21216425 PMC3238379

[ref3] Cargnelutti D. E. Salomón M. C. Celedon V. García Bustos M. F. Morea G. Cuello-Carrión F. D. . (2016). Immunization with antigenic crude protein extracts of *Leishmania* associated with montanide ISA 763 adjuvant induces partial protection in BALB/c mice against *Leishmania* (*Leishmania*) *amazonensis* infection. J. Microbiol. Immunol. Infect. 49, 24–32. doi: 10.1016/j.jmii.2014.01.006, 24662018

[ref4] Casella C. R. Mitchell T. C. (2008). Putting endotoxin to work for us: monophosphoryl lipid a as a safe and effective vaccine adjuvant. Cell. Mol. Life Sci. 65, 3231–3240. doi: 10.1007/s00018-008-8228-6, 18668203 PMC2647720

[ref5] Chroboczek J. Szurgot I. Szolajska E. (2014). Virus-like particles as vaccine. Acta Biochim. Pol. 61, 531–539. doi: 10.18388/abp.2014_1875, 25273564

[ref6] Cid R. Bol J. (2021). Platforms for production of protein-based vaccines: from classical to next-generation strategies. Biomolecules 11:1072. doi: 10.3390/BIOM11081072, 34439738 PMC8394948

[ref7] Coffman R. L. Seymour B. W. P. Lebman D. A. Hiraki D. D. Christiansen J. A. Shrader B. . (1988). The role of helper T cell products in mouse B cell differentiation and isotype regulation. Immunol. Rev. 102, 5–28. doi: 10.1111/j.1600-065X.1988.tb00739.x, 2966762

[ref8] Conde L. Marciel G. Assis G. M. Freire-de-Lima L. Nico D. Vale A. . (2022). Humoral response in leishmaniasis. Front. Cell Infect. Microbiol. 12:1063291. doi: 10.3389/fcimb.2022.1063291 36579347 PMC9791258

[ref9] Coutinho De Oliveira B. Duthie M. S. Alves Pereira V. R. (2020). Vaccines for leishmaniasis and the implications of their development for American tegumentary leishmaniasis. Hum. Vaccin. Immunother. 16, 919–930. doi: 10.1080/21645515.2019.1678998, 31634036 PMC7227727

[ref10] de Souza M. A. da Silva A. G. Afonso-Cardoso S. R. Favoreto Junior S. Ferreira M. S. (2005). Perfil de isotipos de imunoglobulinas e subclasses de IgG na leishmaniose tegumentar americana. Rev. Soc. Bras. Med. Trop. 38, 137–141. doi: 10.1590/s0037-86822005000200002, 15821787

[ref11] Dubie T. Mohammed Y. (2020). Review on the role of host immune response in protection and immunopathogenesis during cutaneous leishmaniasis infection. J. Immuno. Res. 2020:2496713. doi: 10.1155/2020/2496713, 32656269 PMC7320295

[ref12] Ebrahimpoor S. Pakzad S. Ajdary S. (2013). IgG1 and IgG2a profile of serum antibodies to *Leishmania major* amastigote in BALB/c and C57BL/6Mice 12, 361–367. doi: 10.1016/j.imlet.2021.03.011, 23996712

[ref13] Eom G. D. Chu K. B. Yoon K. W. Mao J. Kim S. S. Quan F. S. (2024). Immunizing mice with influenza virus-like particles expressing the *Leishmania amazonensis* promastigote surface antigen alleviates inflammation in footpad. Vaccine 12:793. doi: 10.3390/VACCINES12070793, 39066431 PMC11281337

[ref14] Facciolà A. Visalli G. Laganà A. Di Pietro A. (2022). An overview of vaccine adjuvants: current evidence and future perspectives. Vaccine 10:819. doi: 10.3390/vaccines10050819, 35632575 PMC9147349

[ref15] Facciolà A. Visalli G. Laganà P. La Fauci V. Squeri R. Pellicanò G. F. . (2019). The new era of vaccines: the “nanovaccinology”. Eur. Rev. Med. Pharmacol. Sci. 23, 7163–7182. doi: 10.26355/eurrev_201908_18763, 31486519

[ref16] Fakharzadeh S. Kalanaky S. Hafizi M. Goya M. M. Masoumi Z. Namaki S. . (2013). The new nano-complex, Hep-c, improves the immunogenicity of the hepatitis B vaccine. Vaccine 31, 2591–2597. doi: 10.1016/j.vaccine.2013.03.030, 23583463

[ref17] Ferreras-Colino E. Moreno I. Gortázar C. Sevilla I. Agulló-Ros I. Domínguez L. . (2023). Oral immunization with heat-inactivated *Mycobacterium bovis* reduces local parasite dissemination and hepatic granuloma development in mice infected with *Leishmania amazonensis*. Res. Vet. Sci. 162:104963. doi: 10.1016/j.rvsc.2023.104963, 37517297

[ref18] González M. A. C. Gonçalves A. A. M. Ottino J. Leite J. C. Resende L. A. Melo-Júnior O. A. . (2023). Vaccination with formulation of nanoparticles loaded with *Leishmania amazonensis* antigens confers protection against experimental visceral leishmaniasis in hamster. Vaccine 11:111. doi: 10.3390/vaccines11010111, 36679956 PMC9863486

[ref19] Gupta R. Arora K. Roy S. S. Joseph A. Rastogi R. Arora N. M. . (2023). Platforms, advances, and technical challenges in virus-like particles-based vaccines. Front. Immuno. 14:1123805. doi: 10.3389/fimmu.2023.1123805, 36845125 PMC9947793

[ref20] Husain A. A. Kashyap R. S. Kalorey D. R. Warke S. R. Purohit H. J. Taori G. M. . (2011). Effect of repeat dose of BCG vaccination on humoral response in mice model. Indian J. Exp. Biol. 49, 7–10, 21365989

[ref21] Javadi M. M. Hosseinzadeh M. T. Soleimani N. Rommasi F. (2022). Evaluating the immunogenicity of gold nanoparticles conjugated RBD with Freund’s adjuvant as a potential vaccine against SARS-CoV-2. Microb. Pathog. 170:105687. doi: 10.1016/j.micpath.2022.105687, 35917987 PMC9339102

[ref22] Kotb E. Alkhaldi A. A. M. Saleh A. A. (2021). Host immune response against leishmaniasis and parasite persistence strategies: a review and assessment of recent research. Biomed. Pharmacother. 139:111671. doi: 10.1016/j.biopha.2021.111671, 33957562

[ref23] Limoncu M. E. Balcioglu I. C. Yereli K. Ozbel Y. Ozbilgin A. (1997). A new experimental in vitro culture medium for cultivation of *Leishmania* species. J. Clin. Microbiol. 35, 2430–2431. doi: 10.1128/jcm.35.9.2430-2431.1997, 9276434 PMC229986

[ref24] Marlais T. Bhattacharyya T. Singh O. P. Mertens P. Gilleman Q. Thunissen C. . (2018). Visceral leishmaniasis IgG1 rapid monitoring of cure vs. relapse, and potential for diagnosis of post kala-azar dermal leishmaniasis. Front. Cell. Infect. Microbiol. 8:427. doi: 10.3389/fcimb.2018.00427, 30619774 PMC6300496

[ref25] Mohrs M. Ledermann B. Köhler G. Dorfmüller A. Gessner A. Brombacher F. (1999). Differences between IL-4- and IL-4 receptor alpha-deficient mice in chronic leishmaniasis reveal a protective role for IL-13 receptor signaling. J. Immuno. 162, 7302–7308, 10358179

[ref26] Morillo C. A. Marin-Neto J. A. Avezum A. Sosa-Estani S. Rassi A. Jr. Rosas F. . (2015). Randomized trial of benznidazole for chronic Chagas’ cardiomyopathy. N. Engl. J. Med. 373, 1295–1306. doi: 10.1056/NEJMoa1507574, 26323937

[ref27] Plotkin S. A. (2010). Correlates of protection induced by vaccination. Clin. Vaccine Immunol. 17, 1055–1065. doi: 10.1128/CVI.00131-10, 20463105 PMC2897268

[ref28] Pollet J. Chen W. Strych U. (2021). Recombinant protein vaccines, a proven approach against coronavirus pandemics. Adv. Drug Deliv. Rev. 170, 71–82. doi: 10.1016/j.addr.2021.01.001, 33421475 PMC7788321

[ref29] Powers J. G. Nash P. B. Rhyan J. C. Yoder C. A. Miller L. A. (2007). Comparison of immune and adverse effects induced by AdjuVac and Freund’s complete adjuvant in New Zealand white rabbits (*Oryctolagus cuniculus*). Lab Anim. (NY) 36, 51–58. doi: 10.1038/laban1007-51, 17885664

[ref30] Pulendran B. Arunachalam P. O’Hagan D. T. (2021). Emerging concepts in the science of vaccine adjuvants. Nat. Rev. Drug Discov. 20, 454–475. doi: 10.1038/s41573-021-00163-y, 33824489 PMC8023785

[ref31] Queiroz A. M. V. Oliveira J. W. D. F. Moreno C. J. Guérin D. M. A. Silva M. S. (2021b). VLP-based vaccines as a suitable technology to target trypanosomatid diseases. Vaccine 9:220. doi: 10.3390/vaccines9030220, 33807516 PMC7998750

[ref32] Queiroz A. M. V. Yanshina Y. A. da Silva Rodrigues E. T. Santos F. L. N. Celedon P. A. F. Maheshwari S. . (2021a). Antibodies response induced by recombinant virus-like particles from triatoma virus and chimeric antigens from *Trypanosoma cruzi*. Vaccine 39, 4723–4732. doi: 10.1016/j.vaccine.2021.05.039, 34053789

[ref33] Rostamian M. Sohrabi S. Kavosifard H. Niknam H. M. (2017). Lower levels of IgG1 in comparison with IgG2a are associated with protective immunity against *Leishmania tropica* infection in BALB/c mice. J. Microbiol. Immunol. Infect. 50, 160–166. doi: 10.1016/j.jmii.2015.05.007, 26066544

[ref34] Salih G. F. Awad A. Assumaidaee M. (2025). Pathophysiology of leishmaniasis. Egypt. J. Vet. Sci. 56, 195–203. doi: 10.21608/EJVS.2024.270292.1849

[ref35] Sanchez Alberti A. Bivona A. E. Cerny N. Schulze K. Weißmann S. Ebensen T. . (2017). Engineered trivalent immunogen adjuvanted with a STING agonist confers protection against *Trypanosoma cruzi* infection. NPJ Vaccines 2:9. doi: 10.1038/s41541-017-0010-z, 29263868 PMC5604744

[ref36] Sanchez Alberti A. Bivona A. E. Matos M. N. Cerny N. Schulze K. Weißmann S. . (2020). Mucosal heterologous prime/boost vaccination induces polyfunctional systemic immunity, improving protection against *Trypanosoma cruzi*. Front. Immunol. 11:128. doi: 10.3389/fimmu.2020.00128, 32153562 PMC7047160

[ref37] Santos F. L. N. Zanchin N. I. T. Krieger M. A. Foti L. Celedón P. A. F.. Proteína recombinante ou sintética, sequência de DNA sintético, cassete de expressão, vetor de expressão, célula hospedeira, método de produção da proteína recombinante ou sintética, composição, uso da proteína recombinante ou sintética, kit para diagnóstico da doença de chagas, e, método para diagnóstico da doença de Chagas. 2018. Available online at: https://arca.fiocruz.br/handle/icict/48836 (Accessed August 18, 2018)

[ref38] Seder R. A. Hill A. V. (2000). Vaccines against intracellular infections requiring cellular immunity. Nature 406, 793–798. doi: 10.1038/35021239, 10963610

[ref39] Siclla Godoy M. K. Carnovale S. I. (2024). *Trypanosoma cruzi* infection: strategies for the development of a vaccine. SCT Proc. Interdis. Insight. Innov. 2:351. doi: 10.56294/piii2024351

[ref40] Stewart E. Triccas J. A. Petrovsky N. (2022). Adjuvant strategies for more effective tuberculosis vaccine immunity. Microorganisms 7:255. doi: 10.3390/MICROORGANISMS7080255, 31409028 PMC6724148

[ref41] Vakili B. Nezafat N. Zare B. Erfani N. Akbari M. Ghasemi Y. (2020). A new multi-epitope peptide vaccine induces immune responses and protection against Leishmania infantum in BALB/c mice. Med. Microbiol. Immunol. 209, 69–79. doi: 10.1007/s00430-019-00640-7, 31696313

[ref42] Visciano M. L. Tagliamonte M. Tornesello M. L. Buonaguro F. M. Buonaguro L. (2012). Effects of adjuvants on IgG subclasses elicited by virus-like particles. J. Transl. Med. 10:4. doi: 10.1186/1479-5876-10-4, 22221900 PMC3311067

[ref43] Wang N. Chen M. Wang T. (2019). Liposomes used as a vaccine adjuvant-delivery system: from basics to clinical immunization. J. Control. Release 303, 130–150. doi: 10.1016/J.JCONREL.2019.04.025, 31022431 PMC7111479

[ref44] WHO World Chagas disease day 2024. https://www.who.int/campaigns/world-chagas-disease-day/2024 (Accessed September 12, 2024).

[ref45] Yasmin H. Adhikary A. Al-Ahdal M. N. Roy S. Kishore U. (2022). Host–pathogen interaction in leishmaniasis: immune response and vaccination strategies. Immunol. 2, 218–254. doi: 10.3390/immuno2010015

[ref46] Zhang L. Xu W. Ma X. Sun X. Fan J. Wang Y. (2023). Virus-like particles as antiviral vaccine: mechanism, design, and application. Biotechnol. Bioprocess Eng. 28, 1–16. doi: 10.1007/S12257-022-0107-8, 36627930 PMC9817464

[ref47] Zhao T. Cai Y. Jiang Y. He X. Wei Y. Yu Y. . (2023). Vaccine adjuvants: mechanisms and platforms. Signal Transduct. Target. Ther. 8:283. doi: 10.1038/S41392-023-01557-7, 37468460 PMC10356842

[ref48] Zimmermann P. Curtis N. (2019). Factors that influence the immune response to vaccination. Clin. Microbiol. Rev. 32:e00084-18. doi: 10.1128/CMR.00084-18, 30867162 PMC6431125

